# Population history, genetic variation, and conservation status of European white elm (*Ulmus laevis* Pall.) in Poland

**DOI:** 10.1186/s13595-022-01157-5

**Published:** 2022-09-05

**Authors:** Monika Litkowiec, Magdalena Chudzińska, Anna Pasławska, Małgorzata Pałucka, Czesław Kozioł, Andrzej Lewandowski

**Affiliations:** 1 Kostrzyca Forest Gene Bank, Miłków, Poland; 2 Institute of Dendrology, Polish Academy of Sciences, Kórnik, Poland

**Keywords:** Central-marginal populations, Bottleneck, Microsatellites, DED pandemic, Population structure

## Abstract

**Key message:**

The core populations of the European white elm (*Ulmus laevis* Pall.) located in Poland maintained slightly higher level of genetic diversity compared to the peripheral populations of this species.

**Context:**

The most severe threat to elms is the loss of natural habitat under the pressures of agriculture and forestry as well as urbanization. The reductions in European white elm populations as well as populations of other elm species have also been caused by Dutch elm disease (DED). Previous studies have indicated a low level of genetic variation in *Ulmus leavis* Pall. However, in Poland, the genetic resources and demographic history of *U. laevis* populations remain poorly documented.

**Aims:**

The genetic resources of *U. laevis* in Poland were identified and characterized. Additionally, tests were performed to identify potential bottleneck signatures and effective population sizes of the examined populations.

**Methods:**

Polymorphism was analyzed using a set of six nuclear microsatellite markers (nSSRs) for 1672 individuals from 41 populations throughout the species range in Poland.

**Results:**

(1) A moderate level of genetic variation was found. (2) A low genetic differentiation and lack of population structuring were identified. (3) Evidence of reduction in population size was found as a consequence of severe, past bottlenecks.

**Conclusion:**

The loss of genetic diversity of *U. laevis* probably occurred in their refugia or shortly after the postglacial recolonization. This loss may have been affected by past DED pandemics similar to those seen at present.

## Introduction

The European white elm (*Ulmus laevis* Pall.) is a broadleaved tree whose occupied natural distribution in Europe extends from central France to the Urals. In the northern part of its range, white elm covers only the southern part of Finland; it reaches the southern end of its range in Albania and Bulgaria and grows in several isolated stands in Turkey (Jalas and Suominen 1999; Collin 2003). Across its natural range in Europe, white elm grows mainly in lowlands and sporadically enters mountainous areas along river valleys. Generally, it is more common in eastern Europe than in western Europe (Boratyńska et al. 2015). As the species preferentially occupies lowland areas, its optimal occurrence range covers fertile and moist habitats in river valleys; it is often found in floodplains, is tolerant to humid soils and periodic flooding, and typically occurs in damp, low-lying areas and as a component of riparian forests. In Poland, white elm is one of three native species of elm: the European white elm (*U. laevis* Pall.), wych elm (*U. glabra* Huds.), and field elm (*U. minor* Mill.). The area occupied by elms covers 17,654 ha, i.e., 0.24% of the total forested area. There are only about 1000 ha dominated by elms (Napierała-Filipiak et al. 2014). The vast majority of elm resources are currently formed by white elm. In at least 1/3 of the sites, white elm are of artificial origin. Most often, the species is represented by isolated individuals or by small groups (Napierala-Filipiak et al. 2016). It seldom occurs in the mountains and does not exceed foothill elevations. *U. laevis* can sow and grow under the canopies of old trees, and the species often emerges from dense grass covers. Therefore, in areas covered by white elm, several generations of elm forest may coexist (Filipiak and Napierała-Filipiak 2015).

Today, the most severe threat to elms is the loss of their natural habitat under the pressures of agriculture, forestry, and urbanization. For the last hundred years, European white elm populations and other elm species have also undergone population reductions caused by Dutch elm disease (Brasier 2000), caused by the non-native fungi, *Ophiostoma ulmi* (Buisman) Nannf. and *O. novo-ulmi* Brasier, which are spread by bark beetles of *Scolytus* Geoffroy (Coleoptera, Scolitidae; Brasier 2001). *U. laevis* is the most resistant to infection (Brasier 2001). This disease spread to large areas of Europe, South America, and Asia, causing very high mortality rates, and contributing to the current very dispersed distribution. Genetic changes associated with small, fragmented populations, and increased isolation may limit the evolutionary potential of a species and affect its ability to adapt to new challenges related to climate change as a consequence of lost genetic diversity via random drift (Schaberg et al. 2008). Small, fragmented populations are more prone to adverse effects due to random processes such as the “founder” and “bottleneck” effects (Sork and Smouse 2006).

Recently, special attention has been given to the protection of remaining natural or seminatural riparian forest communities. In many countries, there are places where it has become important to re-naturalize river valleys to restore their natural, economic, and recreational value. The white elm is an extremely important element of these communities. The first step to develop a species protection strategy is to document the level and pattern of its genetic variation. Microsatellite markers of nuclear DNA are widely used in this respect (e.g., Litkowiec et al. 2018; Scotti-Saintagne et al. 2021). Previous research on the genetic structure of *U. laevis* in Europe using different marker systems demonstrated a low level of overall genetic variation and significant differentiation between populations, especially in peripheral populations of the species (Vakkari et al. 2009; Nielsen and Kjær 2010; Fuentes-Utrilla et al. 2014).

Microsatellite markers have been developed and successfully used for many elm species (Whiteley et al. 2003; Collada et al. 2004; Zalapa et al. 2008). However, the degree of *U. laevis* genetic diversity in Poland has not been determined thus far. This study was initiated to shed light on the genetic diversity and structure of many elm populations throughout their central range in Poland. We aimed to answer several questions: (1) Is the level of genetic diversity and genetic differentiation within and among examined populations comparable to other populations from entire natural distribution? (2) Is the observed genetic diversity the result of recent population decline or severe, past population bottlenecks? (3) Is the genetic structure of examined populations as a consequence of postglacial history?

## Materials and methods

### Plant sampling

**Fig. 1:**
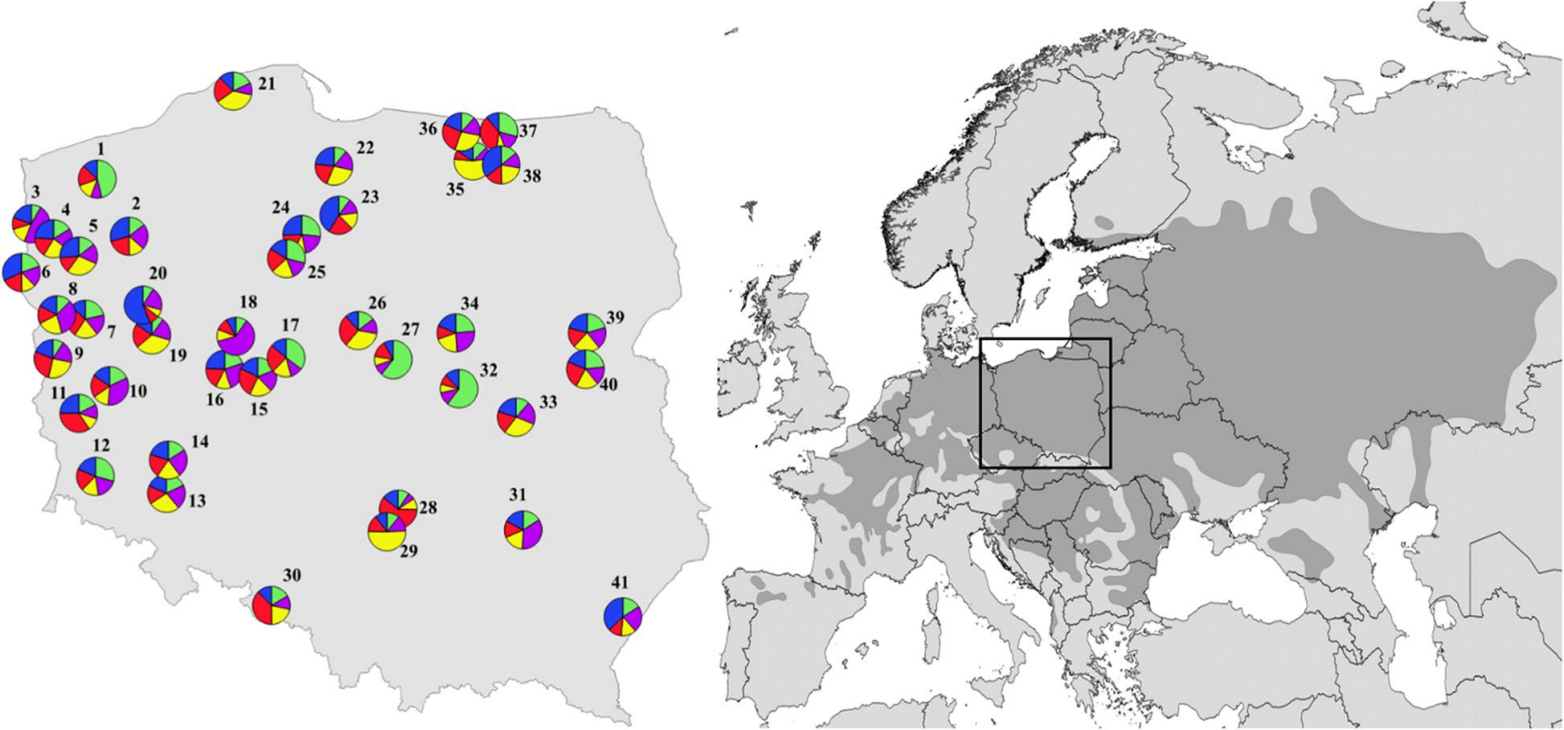
Map of the current natural range of *U. laevis* in Europe (based on the European Forest Genetic Resources programme (EUFORGEN), www.euforgen.org) and the locations of the 41 Polish populations included in this study. The codes representing the populations are listed in Table 1. The proportion of the membership coefficient of each individual in 41 *U. laevis* populations are shown for the inferred number of clusters of 5 (*K* =5), as determined from the STRUCTURE analysis

This study examined 41 *U. laevis* populations from the entire species range in Poland (Fig. 1). The number of specimens sampled from each population ranged from 11 to 50 individuals; a total of 1672 individuals were analyzed (Table 1). Most trees were located near watercourses and lakes in fertile or moderately fertile, moist areas. Almost half of the collected material came from nature reserves. Additionally, most of the areas from which the research material was collected were located in nature protection areas (Natura 2000).

**Table 1:** Site locations, codes, sample sizes, and LBG code of genetic resources of the *U. laevis* Pall. populations studied

No	Code	Name	Sample size	Locality		LBG Code
				Latitude	Longitude	
**1**	WIR	Wiązy Reskie^a^	50	15.3528	53.7844	WZS/10/09
**2**	GRZ	Grądowe Zbocza ^a^	15	15.5707	53.2712	WZS/10/03
**3**	POJ	Podjuchy	50	14.5656	53.3171	WZS/10/02
**4**	TRB	Trawiasta Buczyna ^a^	50	14.7401	53.2798	WZS/10/05
**5**	ZRB	Źródliskowa Buczyna ^a^	37	14.6939	53.2978	WZS/10/04
**6**	OLO	Olszyny Ostrowskie ^a^	46	14.3155	52.9332	WZS/10/06
**7**	LEM	Lemierzyce ^a^	50	14.9078	52.5691	WZS/10/07
**8**	SŁO	Słońsk	31	14.8254	52.5708	WZS/10/08
**9**	URA	Urad	50	14.7028	52.2515	WZS/14/02
**10**	KLE	Klenica	48	15.6799	51.9903	WZS/14/03
**11**	BIE	Bieniów	50	15.2548	51.7142	WZS/14/01
**12**	BRZ	Brzeźnik	50	15.5366	51.1965	WZS/13/03
**13**	WIC	Wilczków	49	16.5163	51.2327	WZS/13/01
**14**	URW	Uroczysko Wrzosy ^a^	50	16.5528	51.3654	WZS/13/05
**15**	CZL	Czeszewski Las ^a^	46	17.5223	52.1342	WZS/09/01
**16**	DWU	Dwunastak ^a^	40	17.5197	52.1641	WZS/09/02b
**17**	SOK	Sokółki ^a^	50	18.2017	52.2816	WZS/09/05
**18**	BIL	Bielawy ^a^	50	17.4672	52.4577	WZS/09/03
**19**	WIL	Wielki Las ^a^	43	16.2538	52.4479	WZS/09/07
**20**	BJL	Buki nad Jeziorem Lutomskim ^a^	50	16.1305	52.6154	WZS/09/06
**21**	WIW	Wielka Wieś	50	17.3474	54.5976	WZS/11/01
**22**	LAM	Las Mątawski ^a^	31	18.8803	53.9602	WZS/15/01
**23**	ROZ	Rogóźno Zamek ^a^	28	18.9521	53.5191	WZS/12/04
**24**	LOP	Łęgi na Ostrowiu Panieńskim ^a^	22	18.4078	53.3563	WZS/12/03
**25**	WIK	Wielka Kępa ^a^	30	18.1880	53.1435	WZS/12/02
**26**	OLR	Olszyny Rakutowskie ^a^	50	19.2396	52.5186	WZS/12/01
**27**	LUS	Luszyn	34	19.7379	52.2654	WZS/06/02
**28**	KRZ	Krzętów	50	19.1957	51.0863	WZS/06/01
**29**	MEL	Mełchów	30	19.6289	50.7626	WZS/02/02
**30**	BAB	Baborów	40	18.0320	50.1159	WZS/02/01
**31**	KLC	Kleczanów	11	21.5265	50.7528	WZS/16/02
**32**	SKU	Skuły	50	20.6729	51.9997	WZS/16/03
**33**	POD	Podłęż ^a^	50	21.4821	51.7391	WZS/17/02
**34**	SZC	Szczypiorno	50	20.6476	52.4860	WZS/17/01
**35**	TUM	Tumiany	40	20.9637	53.9839	WZS/07/04
**36**	GRA	Graniczne	24	21.0787	54.2841	WZS/07/01
**37**	BOB	Bobry	34	21.3484	54.2873	WZS/07/02
**38**	MAK	Mały Kamień	33	21.4564	54.1786	WZS/07/03
**39**	PRZ	Przekop ^a^	42	22.6159	52.3843	WZS/05/02
**40**	KAL	Kaliniak ^a^	49	22.5761	52.3630	WZS/05/01
**41**	STU	Stubno	19	23.0316	49.8933	WZS/04/01

^a^Nature reserve

### DNA extraction, amplification, and genotyping

The total genomic DNA was isolated from approximately 20 mg of leaf tissue using an ISOLATE II PLANT DNA kit (Bioline, London, UK). Suitable markers originally described for *Ulmus* species were tested for their ability to provide repeatable, high-quality results, sufficient polymorphism and unambiguous allele binding. Finally, six polymorphic markers: Ulm19, Ulm2, Ulm3, Ulm6, Ulm9 (Whiteley et al. 2003), and UR188a (Zalapa et al. 2008) were simultaneously amplified in a multiplex reaction using Multiplex Master Mix (Qiagen, Hilden, Germany). The polymerase chain reaction (PCR) program was as follows: 3 min at 94 °C; 30 cycles of 15 s at 94 °C, 90 s at 53 °C, and 2 min 72 °C, and 20 min at 72 °C. The fluorescently labeled PCR products, along with a size standard (GeneScan 600 LIZ, Thermo Fisher Scientific, Waltham, Massachusetts, USA), were separated on an ABI 3500 capillary sequencer (Thermo Fisher Scientific, Waltham, Massachusetts, USA). Alleles were identified based on their size using GeneMapper software (ver. 5.0; Thermo Fisher Scientific, Waltham, Massachusetts, USA), and all variants were checked and approved manually (Litkowiec 2022).

### Genetic diversity and differentiation

The following genetic diversity estimators were computed using FSTAT v 2.9.3 software (Goudet 2001): the number of alleles (A), allelic richness (AR), estimated for the minimum sample size of 11 individuals, observed heterozygosity (H_o_), and unbiased expected heterozygosity (H_e_). The number of private alleles (P_a_) and effective number of alleles (A_e_) were calculated using GenAlEx 6 (Peakall and Smouse 2006). An allele was declared “private” when it was detected only in a particular population and was absent in the other populations.

Microsatellite markers are susceptible to genotyping errors, such as null alleles (Guichoux et al. 2011), and can overestimate the *F*-statistic on account of false homozygotes in populations (e.g., Litkowiec et al. 2018). Therefore, the loci were also tested for the presence of null alleles (N0) using INEST 2.0 software (Chybicki and Burczyk 2009). The multiple sample score test (U test, Raymond and Rousset 1995), implemented in GENEPOP ver. 4.3 (Rousset 2008), was used to determine the significant deviation from Hardy-Weinberg equilibrium (HWE).

The genetic differentiation among populations was assessed using *F*_st_ values with FSTAT v. 2.9.3. Considering the presence of null alleles at all loci, FreeNA software was used to estimate the *F*_st_ values based on the Cavalli-Sforza and Edwards (1967) genetic distance using the Excluding Null Alleles (ENA, F_st_*ENA*) correction method (Chapuis and Estoup 2007). The bootstrap 95% confidence intervals (CI) for the global *F*_st_ values were calculated using 10,000 replicates over the analyzed loci. We also compared the pairwise *F*_st_ and *R*_st_ values to indicate the phylogeographic structure using the SpaGeDi 1.3d program (Hardy and Vekemans 2002; Hardy et al. 2003). *R*-statistics are analogous to *F*-statistics but are based on allele sizes instead of allele identity; *R*_st_ assumes the diversity resulting from genetic drift and mutation processes according to a stepwise mutation model (SMM). The *R*_ST_ values were compared after the allele sizes were permuted using the within loci pR_st_ (permuted *R*_st_, corresponding to *F*_st_). The statistical significance of the alternative hypothesis of *R*_st_ > pR_st_, which suggests that allele size mutations contributed to the population differentiation, was estimated by a permutation (10 000 permutations) test implemented in the SpaGeDi 1.3d program (Hardy and Vekemans 2002).

The genetic structure of the white elm populations was evaluated using the Bayesian clustering method implemented in STRUCTURE ver. 2.3.4 (Pritchard et al. 2000). The assumed parameter sets were admixture allele models with correlated allele frequencies and no prior information about the location of the analyzed population. The Monte Carlo Markov Chain (MCMC) sampling scheme was run for 200,000 iterations with a 100,000 iteration burn-in period; the *K* values ranged from 1 to 41, and 10 independent replications were performed for each *K* value. The optimal *K* value was estimated using the StructureSelector (Li and Liu 2018) which implements the Evanno’s method (Evanno et al. 2005), as well as four alternative statistical measures (Puechmaille 2016). To check for the presence of isolation by distance (IBD, Rousset 1997), a Mantel correlation test (Mantel 1967) was used. The significance of the correlations between the pairwise geographic distances and pairwise genetic distances, measured as *F*_st_/(1-*F*_st_), was tested using 9 999 permutations implemented in GenAlEx 6.

### Demographic history

With NeEstimator 2.01 (Do et al. 2014), the effective population size (N_e_) of each population was estimated via the linkage disequilibrium method (Waples and Do 2008), assuming a random mating model and a critical allele frequency (*P*_cirt_ = 0.02). The 95% confidence intervals (CI_Ne_) were determined with the jackknife method described by Waples and Do (2008).

The examined *Ulmus* populations were tested for evidence of genetic bottlenecks using two methods. For each population, the *M*-ratios (Garza and Williamson 2001) defined as the ratio of *k* (number of microsatellite alleles) to *r* (overall range in allele size, i.e., *M*=*k*/*r*) and Wilcoxon test for heterozygosity excess (Cornuet and Luikart 1996) were calculated using INEST 2.2 software (Chybicki and Burczyk 2009). This analysis was performed using the two-phase mutation (TPM) model with two parameters: the proportion of multistep mutations (pg) and the mean size of multistep mutations (δg). The parameters pg = 0.22 and δg = 0.31 were used as recommended (Peery et al. 2012). The significance of a potential bottleneck was tested using Wilcoxon’s signed-rank test *P* values based on 1,000,000 permutations to obtain approximate values. Also, we used microsatellite data with the approximate Bayesian computation (ABC) method, implemented in DIYABC v 2.0.1 (Cornuet et al. 2014) to analyze the population demographic history. We used three different scenarios to test the changes in population size. Scenario 1 is a constant size population of white elm (Ne constant from past to present); scenario 2 is a population expanded recently, Na (Ne during the expansion, Ne < Na); and scenario 3 consist of a population that still experiencing a bottleneck, Nb (Ne during the bottleneck, Ne > Nb). We pooled all populations into a single sample, and for scenario construction, a total 10,000 simulations were performed to generate the reference table, and all summary statistics included in the DIYABC were used (Cornuet et al. 2014). The posterior probability of each scenario was assessed using logistic approaches (Cornuet et al. 2014). The scenario with the highest posterior probability was selected, and the associated parameters were determined.

## Results

### Genetic diversity and differentiation

All nSSRs were polymorphic, and only 59 alleles were detected in the studied populations, among which 14 were private alleles. The smallest number of alleles (6) was found in the Ulm6 locus, and the highest number (15) was found in both the Ulm3 and Ulm9 loci. Low frequencies of null alleles were found, with an average frequency of 0.014. As the frequency of null alleles did not exceed the threshold (0.2) over which null alleles can result in a significant underestimation of H_e_ (Chapuis and Estoup 2007), all loci were used in the further analyses.

In general, the studied populations were characterized by a moderate level of genetic variation (Table 2). The mean number of alleles (A) was 4.2, ranging from 3.7 in the LAM population to 5.7 in the KLE population. The effective number of allele (A_e_) values was much lower, ranging from 2.1 in the LAM population to 2.6 in the WIR population, with an average value of 2.4. Due to the unequal sizes of the studied populations, the allelic richness (AR) values were calculated by reducing the sizes of all populations to 11 individuals (the size of the KLC population). The mean AR value was 4.0, and the populations were quite homogeneous, with AR values ranging from 3.0 in ZRB and BAB to 3.9 in KLE and PRZ. Private alleles (P_a_) were found in nine populations at very low frequencies (below 10%), with an average frequency of 5%. The largest *P*_a_ number was detected in the BOB population (*P*_a_ = 4), and the POD and KLE populations each had two private alleles. The observed (*H*_o_) and expected (*H*_e_) heterozygosity values ranged from 0.595 (KLE) to 0.675 (BIE) and from 0.529 (LAM) to 0.585 (BIE), respectively. The mean *H*_o_ value (0.641) was higher than the mean *H*_e_ value (0.553), indicating an excess of heterozygotes (*F*_is_ = −0.149). The deviation of genotypic frequencies from Hardy–Weinberg equilibrium (HWE) in all studied populations was not statistically significant in all cases. The pairwise *F*_st_ values ranged from −0.0033 to 0.227, with an overall *F*_st_ of 0.076 (CI95% = 0.053–0.092; *p* < 0.001). The differentiation value was somewhat lower when the null alleles were included, with an F_st_ENA value of 0.074 at a 95% confidence interval (CI95% = 0.055–0.090). This result suggests that null alleles have a nonsignificant influence on the differentiation pattern among populations.

**Table 2:** Parameters used to estimate the genetic structure of the *Ulmus laevis* populations studied, averaged across six nuclear microsatellite loci

CODE	A	Ae	AR_**11**_	Pa	Ho	He	Fis	N0 %
WIR	4.0	2.6	3.8	0	0.635	0.540	−0.139	0.18
GRZ	4.0	2.3	3.6	0	0.639	0.542	−0.186	0.00
POJ	4.0	2.3	3.1	0	0.638	0.540	−0.214	0.00
TRB	4.0	2.3	3.4	0	0.636	0.539	−0.118	1.02
ZRB	4.0	2.3	3.0	0	0.635	0.538	−0.169	0.80
OLO	4.0	2.3	3.3	0	0.638	0.541	−0.135	0.48
LEM	4.0	2.3	3.2	0	0.635	0.538	−0.146	0.33
SŁO	4.0	2.3	3.5	0	0.634	0.539	−0.078	1.53
URA	4.2	2.4	3.3	0	0.637	0.573	−0.101	1.12
KLE	5.7	2.5	3.9	2	0.595	0.573	−0.030	2.90
BIE	4.8	2.5	3.5	1	0.675	0.585	−0.144	2.48
BRZ	4.1	2.4	3.6	0	0.640	0.548	−0.167	2.86
WIC	4.1	2.4	3.8	0	0.640	0.549	−0.035	2.25
URW	4.1	2.4	3.7	0	0.641	0.550	−0.161	0.06
CZL	4.0	2.3	3.8	0	0.648	0.549	−0.069	0.69
DWU	4.0	2.3	3.6	0	0.646	0.549	−0.102	1.91
SOK	4.0	2.3	3.1	0	0.645	0.546	−0.253	0.0
BIL	4.0	2.3	3.3	1	0.650	0.547	−0.178	0.30
WIL	4.0	2.3	3.6	1	0.645	0.548	−0.114	0.91
BJL	4.0	2.3	2.2	1	0.643	0.542	−0.362	0.00
WIW	4.0	2.3	3.6	0	0.635	0.540	−0.164	0.00
LAM	3.7	2.1	3.1	0	0.608	0.529	−0.133	1.39
ROZ	4.0	2.3	3.8	0	0.636	0.541	−0.123	0.56
LOP	4.0	2.3	3.3	0	0.634	0.539	−0.173	0.00
WIK	4.0	2.3	3.8	0	0.638	0.544	−0.118	0.74
OLR	4.0	2.3	3.5	0	0.639	0.545	−0.111	2.91
LUS	4.0	2.3	2.8	0	0.650	0.547	−0.321	1.61
KRZ	3.9	2.3	2.6	0	0.643	0.539	−0.244	0.02
MEL	4.0	2.4	3.7	0	0.645	0.567	−0.110	2.05
BAB	4.0	2.4	3.0	0	0.643	0.552	−0.219	7.40
KLC	4.0	2.3	3.7	0	0.639	0.542	−0.033	2.48
SKU	4.0	2.3	3.6	1	0.642	0.542	−0.166	1.32
POD	4.1	2.3	3.7	2	0.639	0.547	−0.028	0.80
SZC	4.1	2.3	3.4	0	0.638	0.546	−0.177	0.97
TUM	3.9	2.3	3.2	1	0.645	0.543	−0.114	0.51
GRA	3.9	2.3	3.4	0	0.646	0.541	−0.171	0.82
BOB	3.9	2.3	3.5	4	0.645	0.540	−0.181	0.31
MAK	3.9	2.3	3.2	0	0.647	0.543	−0.157	2.28
PRZ	4.1	2.3	3.9	0	0.647	0.554	−0.117	1.33
KAL	4.0	2.3	3.5	0	0.640	0.544	−0.166	0.04
STU	4.0	2.3	3.2	0	0.640	0.544	−0.198	2.20
**Mean**	**4.2**	**2.4**	**4.0**	**0.05**	**0.641**	**0.553**	−**0.149**	**1.21**

*A* average number of alleles, *A*_*e*_ effective number of alleles, *AR* average allelic richness (after rarefaction), *P*_*a*_ number of private alleles, *H*_*e*_ unbiased expected heterozygosity, *H*_*o*_ observed heterozygosity, *N0 (%)* null allele frequency, *F*_*is*_ Wright’s fixation index (all not significantly differ from zero). The population codes are shown in Table 1

The global genetic differentiation based on allele size (*R*_st_ = 0.061; CI95% = 0.046–0.124) was not significantly different from the differentiation that accounted for allele identities (pR_st_ = 0.077, *p* = 0.481), indicating the absence of a geographic structure and that gene flow is high compared with the mutation rate.

### Genetic structure

**Fig. 2:**
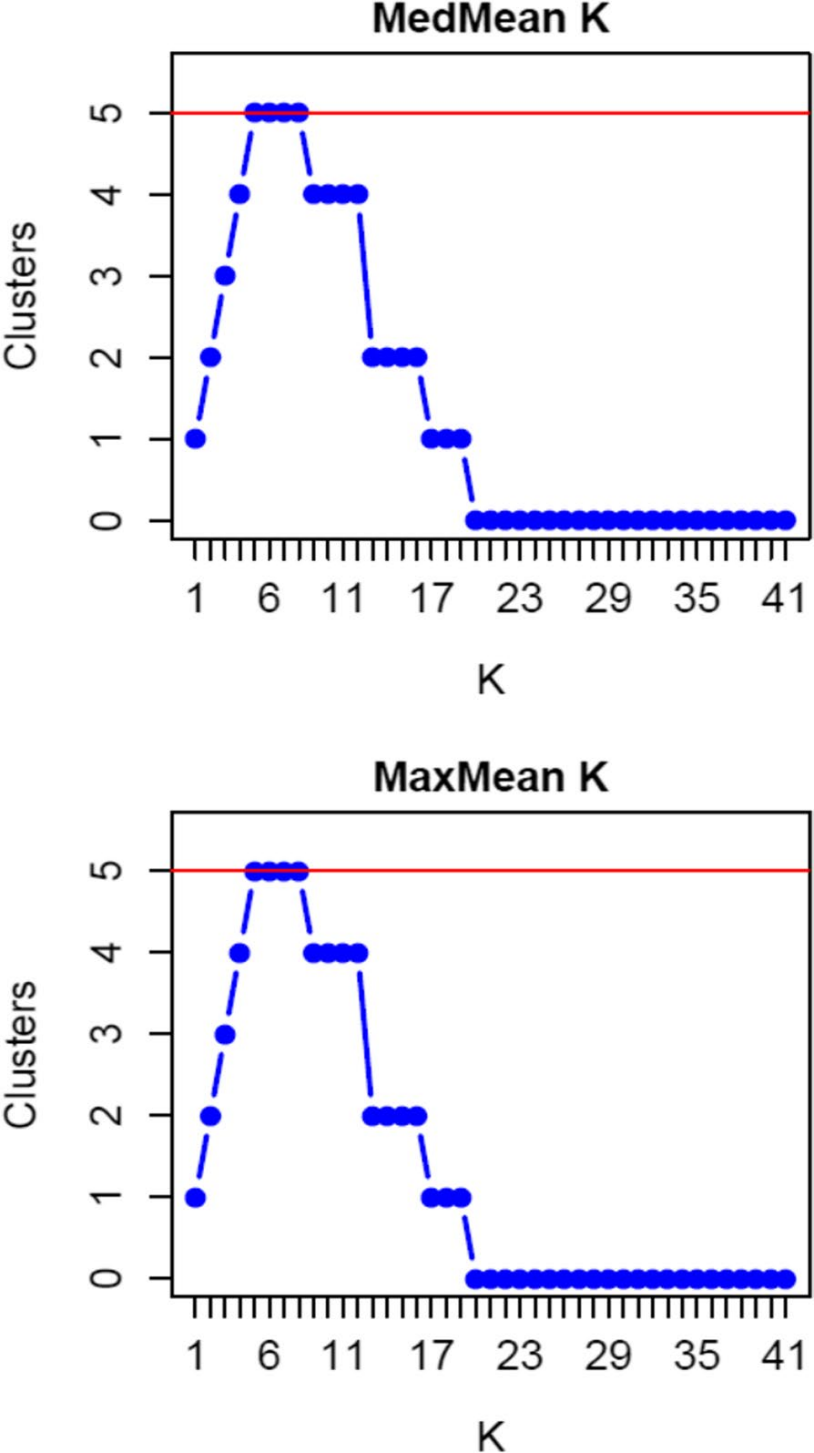
The optimal *K* number indicated by alternative measures MedMean K and MaxMean K applied in STRUCTURE SELECTOR

Grouping and thus finding the optimal number of clusters (*K*) is difficult because the current genetic structure of natural species populations is multifaceted and complex, as a consequence of the demographic, environmental, and historical processes influence (Meirmans 2015). As the Evanno method (delta *K*) did not lead to a biological interpretation for our dataset, we used alternative measures (MedMean and MaxMean) proposed by Puechmaille (2016) to find optimal *K* for our populations. Puechmaille methods were found to be more accurate than delta *K* or mean Ln P(*K*) with unevenly sampled populations, which is our case. The optimal number of clusters was *K* = 5 for all 41 *U. laevis* populations (Fig. 2). However, the proportions of each cluster in the gene pools of the examined populations were comparable and homogenous, except for six populations for which the frequency of one of the four clusters was slightly higher than 0.55 (Fig. 1). Mantel tests of isolation by distance found nonsignificant correlation between the geographical and genetic distance matrices (*R* = 0.002, *p* = 0.328).

### Demographic history and effective population size

The effective population size based on linkage disequilibrium (N_e_LD) varied widely among the white elm populations and ranged from 2.7 (STU) to 360.9 (LEM), an overall harmonic mean of 16.8 (Table 3). The N_e_LD in the 11 white elm populations was lower than the overall harmonic mean N_e_LD. Most Ne confidence intervals overlapped (not a surprise when using few loci) and their interpretation was done with caution. The M-ratios (MRs) were significantly reduced according to the mean MRs derived under mutation-drift equilibrium (Mr_eq_) for all examined white elm populations; this result is strong evidence of a past bottleneck. On the other hand, Wilcoxon’s test for heterozygosity excess (H_e_), performed under the TPM model, indicated recent population reductions in only ten of the analyzed populations (Table 3). Moreover, in the inference of population demographic of Polish populations *U. laevis,* the scenario 2 “recent expansion” was highly favored (posterior probability = 0.481 [CI95 % = 0.4230 - 0.538]) over the scenario 1 “stable population” (posterior probability = 0.325 [CI95 % = 0.2717 - 0.3790]) and the scenario 3 “bottleneck population” (posterior probability = 0.1935 [CI95 % = 0.1449 - 0.2421]). This result revealed that the N_e_ population of *U. laevis* has undergone population size reduction in the postglacial refugia or shortly after the postglacial recolonization. The DIYABC result was in accordance with the result from *M*-ratio which revealed an ancestral and extended declines of population size.

**Table 3:** Genetic bottleneck and estimates of the effective population sizes of *U. leavis*. *He TPM* the excess heterozygosity determined using the TPM mutation model; *MR* the M-ratio, He and MR *p* values, the *p* values were obtained using Wilcoxon’s signed-rank test; *NeLD* the effective population size; *CINe* is shown at the 95% confidence interval. The population codes are listed in Table 1

CODE	He TPM	He ***p*** value	MR	MR ***p*** value	NeLD	CI_**Ne**_
WIR	0.591	**0.046**	0.534	<0.0001	45.1	21–408
GRZ	0.562	0.218	0.486	<0.0001	NA	-
POJ	0.489	0.421	0.425	<0.0001	24.4	12–65
TRB	0.530	0.499	0.469	<0.0001	191.1	41–∞
ZRB	0.513	0.340	0.514	<0.0001	25.1	88–187
OLO	0.540	0.156	0.460	<0.0001	32.1	15–98
LEM	0.556	**0.031**	0.512	<0.0001	360.9	43–∞
SŁO	0.553	0.219	0.475	<0.0001	17.3	7–51
URA	0.578	0.219	0.425	<0.0001	10.2	4–18
KLE	0.579	0.719	0.492	<0.0001	72.5	32–900
BIE	0.591	0.218	0.487	<0.0001	8.9	4–15
BRZ	0.620	**0.015**	0.514	<0.0001	35.7	17–122
WIC	0.615	0.156	0.522	<0.0001	32.9	18–73
URW	0.589	0.156	0.518	<0.0001	47.9	24–164
CZL	0.579	0.500	0.532	<0.0001	19.3	11–34
DWU	0.556	0.422	0.543	<0.0001	12.5	7–23
SOK	0.503	0.218	0.513	<0.0001	18.9	10–41
BIL	0.611	**0.015**	0.464	<0.0001	13.1	6–26
WIL	0.581	0.577	0.484	<0.0001	24.8	13–57
BJL	0.520	0.062	0.402	<0.0001	60.5	10–∞
WIW	0.539	0.281	0.486	<0.0001	19.0	11–35
LAM	0.536	0.109	0.522	0.016	7.3	3–17
ROZ	0.636	**0.015**	0.543	0.017	5.4	3–11
LOP	0.521	0.109	0.505	<0.0001	8.7	3–30
WIK	0.636	**0.031**	0.493	<0.0001	16.9	7–54
OLR	0.609	0.109	0.518	<0.0001	209.5	47–∞
LUS	0.508	**0.031**	0.473	<0.0001	22.5	7–263
KRZ	0.469	0.078	0.469	<0.0001	38.5	12–∞
MEL	0.621	**0.046**	0.457	< 0.0001	25.8	11–146
BAB	0.523	0.156	0.507	<0.0001	9.0	3–27
KLC	0.572	0.218	0.480	<0.0001	NA	-
SKU	0.532	0.499	0.543	<0.0001	51.1	24–239
POD	0.603	0.344	0.541	0.016	12.9	8–20
SZC	0.524	0.421	0.470	<0.0001	9.2	4–16
TUM	0.554	0.218	0.477	<0.0001	262.9	30–∞
GRA	0.575	0.077	0.562	<0.0001	26.2	8–∞
BOB	0.535	**0.001**	0.524	<0.0001	16.8	8–39
MAK	0.585	**0.015**	0.503	<0.0001	16.8	6–59
PRZ	0.617	0.155	0.474	<0.0001	42.5	19–248
KAL	0.549	0.577	0.510	<0.0001	186.5	40–∞
STU	0.527	0.218	0.467	<0.0001	2.7	2–8
**Mean**					**16.8**	

## Discussion

### Genetic variation

Overall, the populations examined in our study showed a moderate level of genetic diversity. This is contrary to the premise that outcrossed, wind-pollinated, widespread temperate trees typically exhibit high levels of within-population genetic diversity and low to moderate levels of among-population genetic differentiation resulting from large populations, extended gene flow, and phenotypic plasticity (e.g., Hamrick et al. 1992; Nybom 2004). Although the Polish populations of European white elm are in the core of the natural range of the species, their level of genetic variation is slightly higher compared to the level of genetic variation maintained by the peripheral populations of this species (Nielsen and Kjær 2010; Venturas et al. 2013; Fuentes-Utrilla et al. 2014; Tamošaitis et al. 2021).

Some of the analyzed nuclear loci were previously used in the study of European white elm in other part of Europe. Out of the set of six loci, four (Ulm2, Ulm3, Ulm9, and UR188a) were also analyzed in Danish (Nielsen and Kjær 2010) and five (Ulm2, Ulm3, Ulm9, Ulm19, and UR188a) in Spanish populations (Venturas et al. 2013). Overall, much more alleles have been found in Poland than in the Spanish and Danish populations. For example, the most variable Ulm3 and Ulm9 loci in Poland each had 15 alleles. In the material from Denmark and Spain, there were only 4 and 3 alleles for the Ulm3 locus and 7 and 6 alleles for the Ulm9 locus, respectively. Presented data suggest that selected nuclear microsatellite loci are polymorphic and thus accurate to infer for level of genetic diversity. In Polish populations of *U. laevis*, genetic diversity parameters, such as the mean number of alleles and observed heterozygosity (*A* = 4.2; *H*_o_ = 0.641), were slightly higher than those in Spanish populations (*A* = 2.6; *H*_o_ = 0.490), Danish populations (*A* = 3.8; *H*_o_ = 0.530), and Norwegian group of trees (*A* = 2.8; *H*_o_ = 0.560) (Nielsen and Kjær 2010; Fuentes-Utrilla et al. 2014). However, as each of these studies analyzed a different number of trees and utilized different microsatellite marker sets, one should exercise caution when comparing results. Notably, in Poland, more than 1 500 *U. laevis* individuals were analyzed, in contrast to the 91 and 110 trees analyzed in Denmark and Spain, respectively. Moreover, differences in genetic variation levels may result from the locations of the studied populations. The Danish and Spanish *U. laevis* populations are located along the edge of the natural range of this species. Another study based on different system markers such as isozymes pointed out a low level of genetic diversity in marginal Finnish *U. laevis* populations (Vakkari et al. 2009). Our findings indicate that *U. laevis* shows lower genetic diversity at nSSRs compared to congeneric species such as *U. glabra* (Martín del Puerto et al. 2017; Chudzińska et al. 2018; Tamošaitis et al. 2021) and *U. minor* (Buiteveld et al. 2016; Zebec et al. 2016; Chudzińska et al. 2019; Tamošaitis et al. 2021).

In our study, a low to moderate level of genetic differentiation was found among the studied *U. laevis* populations, with and without adjusting for null alleles (*F*_st_ = 0.076 versus *F*_st_Null= 0.074, *p* < 0.01). Similarly, Whiteley (2004) found low differentiation levels among five Central and Northeastern European populations. The overall population differentiation was lower than that observed among Spanish populations (*F*_st_ = 0.155; Fuentes-Utrilla et al. 2014). These differences may have resulted from greater gene flow between the Polish elm populations located in a smaller area than the Spanish populations, which may have prevented increased genetic differentiation among populations. Moreover, in this study, the global genetic differentiation estimated based on allele sizes (*R*_st_) was lower than that of the pR_st_ analog to *F*_st_, indicating that random genetic drift was more important than mutation in causing the observed differences among the studied *U. laevis* populations in Poland.

The Bayesian clustering methods indicated that five clusters (*K* = 5) most likely provided representations of the overall genetic structure of the analyzed *U. laevis* populations. Generally, the gene pools of the analyzed populations were genetically homogeneous. The studied populations had comparable gene pool compositions except for a few populations, where one of five clusters was dominant. The non-geographically structured gene pool of Polish *U. laevis* populations was confirmed by the non-significant Mantel test (*R* = 0.002, *p* =0.328). The current genetic divergence pattern of *U. laevis* in Europe, including in Poland, is the result of Quaternary climate change leading to a reduction in population sizes and the long-term isolation of populations during glacial-interglacial cycles and postglacial migration (Hewitt 2000). The observed genetic structure pattern implies the occurrence of free gene exchange among populations and that they probably share a common postglacial history. The investigation of postglacial history of *U. laevis* in Europe, using chloroplast DNA markers (cpDNA) identified three cpDNA haplotypes (A, B, and C) which are characteristic for potential glacial refugia for white elm (Whiteley 2004). The haplotype A was found with high frequency from France to Northwest Russia of the natural range distribution of *U. laevis.* The other two haplotypes are very rare. The haplotype B was found in southern France while the haplotype C was found in the Balkans and Southwest Russia. The presence of both haplotypes A and C in Russia indicates a core Russian glacial refuge from which current white elm populations have originated by postglacial expansion. However, the southern distributions of haplotypes B and C could indicate additional refugia for white elm, but probably postglacial recolonization from these areas was limited (Whiteley 2004). It can be speculated that the *U. laevis* entered to the territory of Poland from the Russian or the Balkan refuge, or Poland, was under the range of both refugial areas. The hypothesis that *U. laevis* migrated to Poland from two refugia Russian and Balkan is probably true because we observed the heterozygosity excess in all of the studied populations as a consequence of the mixing of two previously isolated populations (“isolate-breaking” effect) (Wahlund 1928). These hypotheses should be verified using cpDNA markers. Moreover, Bayesian analysis of population structure of *U. laevis* performed by Fuentes-Utrilla et al. (2014) showed the differentiation between Iberian/SW France and Central Europe core distribution of this species. The pattern of genetic diversity of Spanish populations is not consistent with the pattern of presence in Central Europe. The authors concluded their results stating that Spanish populations of *U. laevis* may represent relict populations of an Iberian glacial refuge.

### Demographic history

A bottleneck is a factor that can negatively influence the genetic structure of natural populations due to a decline in population sizes increasing the level of inbreeding and reducing the level of genetic diversity; thus, bottlenecks threaten the sustainability of populations in the short and long terms. On the other hand, the life history traits that are common to long-lived forest trees, such as long-distance pollen and seed dispersal, overlapping generations and longevity, may protect populations against the effects of sharp population declines over the short term. In this study, the specific tests used to examine the bottleneck effect with microsatellites yielded interesting results, with the calculated *M*-ratios test suggesting bottlenecks in all populations; however, the heterozygosity excess test performed with the TPM model showed evidence of bottlenecks in only ten of the analyzed populations. Tests that are based on heterozygosity using a given number of alleles are better able to identify recent, less-severe bottlenecks. Recently, bottlenecked populations show excess heterozygosity relative to that expected based on the number of alleles. In contrast, the *M*-ratio is smaller in a bottlenecked population than in an equilibrium population. Moreover, the *M*-ratio test is more powerful at detecting ancestral and extended declines than the heterozygosity excess test (Williamson-Natesan 2005). As the recovery time of the *M*-ratio is longer than that of the heterozygosity excess test, the low *M* value obtained reflects older and more severe reductions in the sizes of the studied populations (Garza and Williamson 2001; Williamson-Natesan 2005). Thus, the low *M*-ratio values obtained for all studied *U. laevis* populations are likely to be a consequence of genetic decline during postglacial recolonization. Our results indicated that the loss of *U. laevis* genetic diversity probably occurred in their refugia or shortly after their postglacial recolonization. This assumption was confirmed by the result of analysis DIYABC, where the best fit scenario to our data probably indicted the reduction size of the ancestral population. Another investigation also showed a signature of historical bottlenecks after Holocene migration in Spanish *U. laevis* populations (Whiteley 2004; Fuentes-Utrilla et al. 2014). Similarly, relict *U. glabra* populations from the Iberian Peninsula experienced historical reductions in their population sizes (Martín del Puerto et al. 2017).

The genetic bottleneck phenomenon is largely related to the *N*_e_ and serves as a warning sign for the conservation status of a given population (Frankham 2005; Luikart et al. 2010). The assumed value of 50/500 proposed by Franklin (1980) has become an essential indicator in conservation genetics. Based on this theory, *N*_e_ = 50 is sufficient to prevent inbreeding depression in the short term (over five generations), whereas *N*_e_ ≥ 500 is appropriate for securing long-term viability because the population can maintain a balance between genetic drift and mutation, thereby retaining its evolutionary potential. The Polish *U. laevis* populations studied herein are characterized by low population sizes with a mean *N*_e_ value of 16.8. Moreover, only eight out of forty-one (20%) examined populations exhibited *N*_e_ values over 50. Based on these assumptions, the studied populations probably maintain a low evolutionary potential. However, field observations of Polish *U. laevis* populations indicated that they are in good condition despite their moderate level of genetic diversity and low effective population size.

### Loss of genetic variation and Dutch elm disease

In Poland, the maximum spread of *Ulmus* in the Holocene started at approximately 6000 B.P. from the southeast direction, before the advent of the Neolithic people, and the proportion of *Ulmus* in the overall forest stand composition was higher than 10% (Ralska-Jasiewiczowa et al. 2003). Then, approximately 5000 B.P., a rapid decrease down to 2% occurred in the proportion of *Ulmus* in the forest stands. Undoubtedly, settlement activities and regional climate change contributed to reduced forest cover in areas where *Ulmus* occurred (Ralska-Jasiewiczowa et al. 2003). However, according to many authors, the rapid reduction rate of *Ulmus* corresponds better with the pathogenic hypothesis caused by cyclical Dutch elm disease pandemics (Girling and Greig 1985; Ralska-Jasiewiczowa et al. 2003; Caseldine and Fyfe 2006). In Poland, the last pandemic was first recorded in Katowice in 1927 and then in northern Poland and Warsaw in 1932 and 1935, respectively. In the 1950s and 1960s, the disease was reported in all parts of the country and caused substantial losses in urban green areas, roadsides, and forests (Mańka 2005). Compared to other tree species with similar life history, the *U. laevis* populations maintained a strongly reduced level of genetic variation and low genetic differentiation. For example, black poplar, which occupies habitats similar to those of *U. laevis*, still maintains a high level of genetic variation and a low level of genetic differentiation (Lewandowski and Litkowiec 2017; Wójkiewicz et al. 2019; Wójkiewicz et al. 2021).

In the case of a species having a high level of genetic variation, the random loss of various alleles as a result of the recent pandemic should increase the level of interpopulation variation; however, we did not observe this in our study. Moreover, only a few populations displayed “private” alleles, with very low frequencies. Thus, it appears that despite the high mortality rate of white elm during the last pandemic, the species lost a small number of alleles. This may indicate that the Polish elm populations that existed before the last pandemic were homogeneous with a low level of genetic variation, similar to that expressed by the species today. Additionally, some other studies have suggested that there is no evidence of genetic diversity losses in elm populations as a consequence of the last pandemic (Nielsen and Kjær 2010; Brunet et al. 2016; Buiteveld et al. 2016). It is possible that elm populations lost most of their genetic variation during previous pandemics. As our research shows, all analyzed populations have experienced severe reductions in their *N*_e_. It cannot be ruled out that the loss of genetic variation in white elm already took place in their refugia; therefore, more extensive research is needed involving populations from other parts of Europe.

## Conclusion

The *U. laevis* populations examined appeared to maintain a moderate level of genetic variation and low genetic differentiation and no evidence of genetic population structuring. Our results indicated demographic processes such as reduced population sizes via past bottlenecks. We speculated that the loss of genetic variation in *U. laevis* probably occurred in their refugia or shortly after their postglacial recolonization. This hypothesis should be confirmed by additional investigation using cpDNA and nSSR markers. Also, a much more detailed sampling of the Russian would be extremely valuable.

Despite the pandemic and moderate genetic diversity, white elm individuals are still quite numerous in Poland. However, in white elm populations, very significant reductions in the numbers of individuals have taken place, and most populations have low effective population sizes. In Poland, a twofold increase has occurred in the area of forest stands dominated by elms in the last 50 years (Napierala-Filipiak et al. 2016), mostly consisting of areas with white elm individuals that are least prone to the disease. *U. laevis* often occurs in legally protected areas, such as nature reserves, or in areas covered by the Natura 2000 network. Therefore, we currently do not see any urgent need for ex situ or in situ conservation action. We propose that the most valuable populations with high effective population sizes, such as TRB, LEM, OLR, TUM, and KAL, be considered candidates for dynamic conservation units (DCUs) in the European information system on forest genetic resources (EUFGIS; http://www.eufgis.org/) conservation network and subjected to continuous monitoring.

## Data Availability

The datasets generated and analyzed during the current study are available in the RepOD repository, 10.18150/LLHKEX.
